# Barriers to Electronic Patient-Reported Outcome Measurement Among Patients with Cancer and Limited English Proficiency

**DOI:** 10.1001/jamanetworkopen.2022.23898

**Published:** 2022-07-22

**Authors:** Elena Garcia Farina, Jessi Rowell, Anna Revette, Ellana K. Haakenstad, Jessica L. F. Cleveland, Rachel Allende, Michael Hassett, Deborah Schrag, Nadine J. McCleary

**Affiliations:** 1 Medical Oncology, Dana-Farber Cancer Institute, Boston, Massachusetts; 2 University of Virginia, Charlottesville; 3 Survey and Data Management Core, Dana-Farber Cancer Institute, Boston, Massachusetts; 4 Information & Analytics, Dana-Farber Cancer Institute, Boston, Massachusetts; 5 Social Work, Dana-Farber Cancer Institute, Boston, Massachusetts

## Abstract

**Importance:**

Often electronic tools are built with English proficient (EP) patients in mind. Cancer patients with limited English proficiency (LEP) experience gaps in care and are at risk for excess toxic effects if they are unable to effectively communicate with their care team.

**Objective:**

To evaluate whether electronic patient-reported outcome tools (ePROs) built to improve health outcomes for EP patients might also be acceptable for LEP patients in the context of oral cancer-directed therapies (OCDT).

**Design, Setting, and Participants:**

This qualitative study was conducted at a single National Cancer Institute–designated comprehensive cancer center. In 2019, English-speaking and Spanish-speaking LEP patients with cancer receiving oral chemotherapies were recruited to participate in a qualitative focus group examining patient attitudes toward ePROs and electronic tools that are used to manage adherence and symptoms related to oral therapies. Six focus groups were held for EP patients and 1 for Spanish-speaking LEP patients. LEP was defined as patients who self-identified as needing an interpreter to navigate the health care system. Data analysis was performed April through June of 2019.

**Exposures:**

Enrolled patients participated in a focus group lasting approximately 90 minutes.

**Main Outcomes and Measures:**

The perspectives of patients with cancer treated with oral chemotherapies on integrating ePROs into their care management.

**Results:**

Among the 46 participants included in the study, 46 (100%) were White, 10 (22%) were Latinx Spanish-speaking, 43 (93%) were female, and 37 (80%) were aged at least 50 years or older. Among the 6 focus groups with 6 to 8 EP patients (ranging from 6 to 8 participants) and 1 focus group with 10 Spanish-speaking LEP patients, this qualitative study found that EP and LEP patients had different levels of acceptability of using technology and ePRO tools to manage their OCDT. EP patients felt generally positive toward OCDT and were not generally interested in using electronic tools to manage their care. LEP patients generally disliked OCDT and welcomed the use of technology for health management, particularly when addressing gaps in symptom management by their oncology clinicians.

**Conclusions and Relevance:**

Although most electronic interventions target EP patients, these findings reveal the willingness of LEP patients to participate in technology-based interventions. Expanding ePROs to LEP patients may help to manage gaps in communication about treatment and potential adverse events because of the willingness of LEP patients to use ePRO tools to manage their health. This qualitative assessment is a strategic step in determining the resources needed to narrow the digital health gap and extend the value of PROs to the LEP oncology population.

## Key Points

**Question:**

Should electronic patient-reported outcome tools (ePROs), built for patients who are English proficient (EP), be implemented for Spanish-speaking patients with limited English proficiency (LEP), particularly in the context of oral cancer-directed therapies (OCDT)?

**Findings:**

This qualitative study of 46 participants found that EP and LEP patients have different levels of acceptability of using technology and ePRO tools to manage their OCDT. EP patients felt generally positive about OCDT and were largely uninterested in managing their care with electronic management tools and ePROs, while LEP patients generally disliked OCDT and welcomed the use of electronic tools including ePRO reporting.

**Meaning:**

These findings suggest that technology-based interventions built for EP patients may also be useful for LEP patients.

## Teaser

This qualitative study investigates patient attitudes toward electronic patient-reported outcome tools as a component of care management for a diverse patient group with English proficiency (EP) and limited English proficiency (LEP) using oral cancer-directed therapies.

## Introduction

More than 60 million people in the United States speak a language other than English at home. The 40% that report that they speak English less than “very well”^1^ are considered to have limited English proficiency (LEP). Although there is no specific data on the proportion of patients with LEP diagnosed with cancer, given that more than 8% of the US population are LEP and more than 0.5% of the US population are diagnosed with cancer,^2^ we estimate that more than 8% of those 1 800 000 diagnosed with cancer annually in the US are LEP. The LEP population faces unique barriers to accessing oncology care, such as difficulty communicating with clinicians and a lack of information in their primary language. Consequently, LEP patients may experience delays in oncology referral, treatment, lower enrollment in cancer clinical trials, and more interruptions to cancer care due to unplanned emergency department (ED) visits, or longer hospitalizations.^3^

Patient-reported outcomes (PROs) have demonstrated benefits in treatment outcomes, survival, and health-related quality of life in the care of patients who are English proficient (EP).^4^ Oftentimes technology tools are built with EP patients at the center of them. This can demonstrate challenges at the time of scaling them to other populations, such as LEP patients. PROs have the potential to address LEP patient barriers to accessing oncology care. PROs are data that patients report directly about their symptoms, experiences with care, health-related quality of life, and other medical measures without interpretation from their clinical care teams. They have become standard tools in the evaluation of treatment efficacy^5^ and can contribute to improvements in symptom management and survival.^6^ PROs offer promising opportunities for integration in clinical practice, specifically in relation to individualized treatments. PROs are widely endorsed for use in clinical trials and standard clinical care by organizations such as the European Medicines Agency,^7^ the International Consortium for Health Outcomes Measurement,^8^ the US Centers for Medicare & Medicaid Services,^9^ the US National Institute of Health,^10^ and the US Food and Drug Administration.^11^ Some of the benefits of PROs in oncology care include increased symptom awareness, improved communication with patients and among the care team, and faster and more efficient consultations.^6^ The success of PROs is contingent on effective administration and adoption,^6^ which is in itself contingent upon effective communication between patients and clinicians. The growing interest and feasibility of using electronically reported PROs (ePROs) are due in part to the increasing integration of technology in health care and to patients’ willingness to use innovative applications to manage their health.^6^

Recognizing the impediments to cancer care faced by the LEP population, and that ePROs are associated with better outcomes and a reduction in resource use, we sought to understand the opportunities to improve these tools to address the needs of LEP patients. Given that Spanish is the most spoken language other than English in the US, and that it’s spoken by 9.1% of people in Massachusetts,^12^ we decided to focus on this population. To this end, we conducted a qualitative study to explore potential facilitators to implementing ePROs for Spanish-speaking and English-speaking patients diagnosed with cancer. We also investigated how to best implement an oral chemotherapy self-management tool at the Dana-Farber Cancer Institute (DFCI) in Boston, Massachusetts.

## Methods

We conducted an exploratory qualitative study to identify patient attitudes within the context of their oral cancer-directed therapy (OCDT) toward the use of the internet and the health care system’s patient portal to communicate with their care team, access health information, manage oral medications, and complete ePRO questionnaires. We additionally inquired about the utility of a portal-based tool for self-management of oral therapies. This study was conducted for quality improvement under the DFCI Patient Reported Data Program and was therefore not reviewed by the Dana-Farber/Harvard Cancer Center institutional review board.

We recruited both EP and LEP patients aged at least 18 years seeking care at DFCI, who had been prescribed oral therapies for at least one treatment cycle in the summer of 2019 by a medical oncology clinician in the Gastrointestinal Cancer or Breast Oncology Centers. Given the limited number of Spanish-speaking patients who were prescribed oral chemotherapy, the LEP focus group was expanded to include any oral prescription for cancer. All patients were between the ages of 50 and 69 years. All EP patients meeting the inclusion criteria were contacted by the study team to participate. Spanish-speaking LEP patients (those who self-identified as requiring an interpreter to navigate the health care system) were identified using a convenience sample from a Spanish-speaking patient support group. All participants met on the DFCI Longwood campus and were compensated with a $25 Target gift card, lunch, and free parking for their time.

In total, 7 focus groups (6 EP, 1 LEP) were conducted. There were 6 to 8 participants in each EP group, and 10 participants in the LEP group. Although the exact sample size was not predetermined, we anticipated that 4 focus groups would be sufficient to achieve code saturation. Our sampling strategy focused on generating sufficient data across a diverse population to better understand key dynamics associated with OCDT experiences. Participants were grouped by demographics and cancer type; focus group characteristics are detailed in Table 1. The first group consisted of patients with breast cancer who were younger than 50 years of age. In the second group, there were patients with breast cancer ranging from 50 to 70 years of age. In the third group, there were patients with breast cancer aged at least 70 years. In the fourth group, there were patients with gastrointestinal cancer younger than 50 years of age. In the fifth group, there were patients with gastrointestinal cancer ranging from 50 to 70 years of age. In the sixth group, there were patients with gastrointestinal cancer aged at least 70 years. The seventh group consisted of LEP patients with cancer who spoke Spanish.

**Table 1.** Focus Group Categories

Group No.	Language	Type of cancer	Age, y
1	English	Breast	<50
2	English	Breast	50-70
3	English	Breast	>70
4	English	GI	<50
5	English	GI	50-70
6	English	GI	>70
7	Spanish	Breast and GI	Any

Abbreviation: GI, gastrointestinal.

The moderator explained the objectives of the focus group, the anonymity of comments, and obtained verbal consent from each participant. The LEP focus group was moderated in Spanish. The discussions lasted approximately 90 minutes. The moderator guide in English can be found in the eAppendix in the Supplement. Each focus group discussion consisted of an open dialogue prompted by the moderator’s questions about experiences with oral therapies, care team communication, the use and perspective on technology for health care management, and an evaluation of patients’ reactions to the idea of a mobile application to help improve treatment adherence and their experience with the care team. Patients were then given a handout that outlined 7 different subjects that could be covered in a medication management app. They were asked to rank the subjects in order of importance. The subjects listed were (1) activity tracker; (2) medication schedule with reminders; (3) medication tracking system; (4) symptom reporter; (5) symptom educational library; (6) communication tool; and (7) calendar for medical appointments.

### Data Analysis

The focus groups were recorded and professionally transcribed; the Spanish transcription was translated into English for the analysis. Thematic analysis was done using NVIVO (QSR International) software during coding and analysis. Transparency and open dialogue were prioritized in the research process and the interdisciplinary team reviewed and discussed each step in the project development, implementation, coding, and analysis. Whereas a single qualitative expert conducted the coding, it was through our combined knowledge and perspectives that the analytical materials—the moderator guide, codebook, and analytic summaries—incorporated a breadth of experiences and perspectives. Data analysis was performed in the summer of 2019.

Inductive and deductive dynamics were incorporated into the coding and analysis process. Initial descriptive coding was guided by the questions posed in the moderator guide. These prefigured codes were then combined with inductive codes identified using open coding. A finalized refined coding structure was then collaboratively and iteratively developed, then applied to all interview transcripts. The analysis process included developing code summaries and looking within and across codes to identify key patterns, contexts, and themes associated with the patient experiences and perspectives of OCDT. The focus groups were used as formative research, and therefore the study team determined that aiming for code saturation would be sufficient to identify the main domains and most salient patient experiences needed to inform future research. Representative quotes for each theme were presented to the team, and the first author (in collaboration with the team) selected specific representative quotes for inclusion in the manuscript, which are found in Table 2.

**Table 2.** Key Focus Group Themes and Patient Quotes

Key themes	Group	Summary	Patient quotes
Perspectives on OCDT	EP	Generally positive perspectives toward OCDT	“When I found out I was on oral therapy, I was glad that I wasn’t getting intravenous therapy.”
			“The convenience, I suppose.”
			“Just living my life, take it at home, go on with your life.”
	LEP	Largely negative perspectives toward OCDT	“I had the most horrible experience of my life.”
			“Why are you making me take this if [it’s so harmful].”
			“I took it for 5 years but it’s very harmful.”
Source of OCDT information	EP	Participants received information from different sources: medical clincians, pharmacists, online sources, OCDT literature/instructions, peers	“I actually went and met with one of the nutritionists here.”
			“When the doctor explained it to me just in an office visit, I was completely confused. So, it was really great the pharmacist had called and just walked me through it, every single step.”
			“I think you need to have a variety of things to access.”
	LEP	Participants received information about side effects from their clinicians	“I asked Dr [last name] that I wanted to go off that medication […] she said no, that if there was any cancer left in my body it would go away with this medication. That I had to follow through with it. But it was such a bitter experience.”
			“The doctor tells you what is going to happen to you.”
			“He [the doctor] sent me along with the pills, a list with all of the horrible side effects. These were 80-something side effects. But they were in English and since I don’t speak English…”
Use of technology	EP	Mixed levels of regular technology use and openness to using electronic tools to manage their health care	“I don’t use—I don’t do anything.”
			“Yeah, I’m always on my computer.”
			“I put all my appointments on my notes.”
			“It was just one more thing to do. I mean, why not just wait until we get there, and we tell them?”
			“It never seemed like they got the information from those [questionnaires] before you went in.”
	LEP	Mixed levels of regular technology use. Openness to using electronic tools to manage their health care	“[Using a computer] is fine for me.”
			“[Using a computer] is too complicated and I forget things.”
			“I only use [the phone] to make and receive phone calls but that is it.”
			“I use [the computer] and I love all of that. I pay my bills there.”
			“Oh, I would like to have that [referring to the hospital’s patient portal application].”
Reaction to mobile application idea	EP	Mixed reactions, with most participants being largely uninterested	“I don’t think I would need it.”
			“Not necessary.”
			“I don’t think I would use it.”
			“I think it would be helpful to have. And I also wonder if that data could be used in—be aggregated so that it could maybe show trends.”
	LEP	Generally positive reactions, as long as there was a navigator. Participants described the idea of an app and navigator as important, wonderful, convenient, and beneficial	“Wonderful.”
			“You learn more.”
			“It’s very convenient.”
Care team experience	EP	Overall positive experience. Two individuals described situations in which they were hesitant to communicate	“Mine is spot on” [in reference to the care team understanding the patient's experience]
			“I think the response here is amazing.”
			“No hesitation on my part to ask questions or interrupt and say things or whatever or ask for a further explanation.”
			“And I know they can’t always be at their desk, I guess, but I find that it’s frustrating because I usually have a question, I need answers.”
	LEP	Overall positive experience. Direct communication often focused on side effects. Some participants emphasized the importance of direct communication with the team, and one perceived their questions to be unwelcomed by their medical team	“The doctor tells you what is going to happen to you.”
			“They never explained what they were going to give me.”
			“Yes, the care here is excellent.”
			“I feel that they ended up hating me because I would call them very often.”

Abbreviations: EP, English proficient; LEP, limited English proficient; OCDT, oral cancer-directed therapies.

## Results

Among the 46 participants included in the study, 46 (100%) were White, 10 (22%) were Latinx Spanish-speaking, 43 (93%) were female, and 37 (80%) were aged at least 50 years or older.

### EP Focus Groups

Key themes expressed by participants who are English proficient are detailed in Table 2. EP participants had generally positive perspectives on OCDT, as it was seen as more convenient than intravenous therapy, and for several participants, the side effects were less severe on OCDT compared with infusions. However, there were some areas of concern, such as side effects, cost and insurance, worrying about food restrictions with OCDT, and the size and smell of the pills. They had various sources of information about OCDT, including medical clinicians, pharmacists, online sources, OCDT literature, and peers.

Most patients regularly used a computer and/or smartphone but were largely uninterested in symptom reporting through ePROs. However, there was a recognition that interest in an ePRO tool might vary, and use would probably vary depending on an individual’s personal situation and preferences. Of the 7 subjects that could be incorporated in a medication management app, patients ranked the symptom reporter as the most important item, followed by the care team communication tool; EP patients ranked the activity tracker, medication schedule with reminders, and symptom educational library as the least important items.

EP patients were concerned about the frequency of symptom reporting questionnaires through an ePRO tool (some said that once a week would be okay, some said once a month; most agreed that daily questionnaires would be too much). They expressed their irritation when clinicians did not appear to have read their responses on ePRO tools in the past. Their reasoning for not wanting to engage in electronic tools to manage their health varied but included the difficulty of constantly focusing on their illness, being too busy, finding it too impersonal, and simply not needing it.

### LEP Focus Group

Key themes expressed by participants who were Spanish speaking with LEP are detailed in Table 2. LEP patients described a lack of explanation from their clinicians about OCDT and the side effects associated with their treatment. This resulted in a general dislike and fear of oral therapies. Respondents shared concerns about potential treatment-related symptoms. Most respondents relied on physicians for treatment teaching, with a few citing independent research using family networks or the internet to obtain information.

Most patients used a computer and/or smartphone regularly and were interested in the use of technology to manage their health. They were very welcoming of the idea of electronically self-reporting their symptoms as long as there was support from a clinician to troubleshoot issues with an app. They asked for all communication and questions about symptom management to be in Spanish and described electronic tools as important and beneficial. Of the 7 subjects that could be incorporated in a medication management app, patients ranked the communication tool and calendar for medical appointments as the most important items and the activity and medication trackers as the least important items.

Patients emphasized the importance of direct communication with the care team when there was an issue with their treatment, most often associated with side effects, but some perceived their questions to be unwelcomed by their care team at times. Additionally, patients described some experiences in which there was insufficient information about their treatment or a moment when their concerns were downplayed by clinicians. These encounters provide an opportunity for improved communication, such as an application or tool integrating ePRO collection with the electronic health record to provide access to all clinical information in one location, allowing for timely correspondence with the care team.

## Discussion

Our findings suggest that EP and LEP patients have different levels of acceptability of using technology and ePRO tools to manage their OCDT. EP patients felt generally positive about OCDT and were generally unenthused about the idea of managing their care with electronic management tools and ePROs. LEP patients generally disliked OCDT but were receptive to the use of electronic tools including ePRO reporting. The findings from the LEP focus groups reveal an opportunity to support patient-clinician communication with electronic tools and ePROs. An electronic treatment management tool would provide an additional mode for physician-patient exchange of information. Integrating ePROs into such a tool would be particularly helpful, as it would provide a standardized and validated record of patient symptoms to manage them. Our results indicate that even a tool built for EP patients may have substantial acceptability among LEP patients, particularly if language concordance is achieved. Where some might exclude LEP patients from technology-based interventions because of perceived difficulties in achieving language concordance, our findings support the inclusion of LEP particularly as an opportunity to address persistent gaps in cancer care for LEP patients.

Several studies have recommended the implementation of PROs and other communication tools to facilitate symptom reporting for LEP patients.^13,14^ Hirsh et al^14^ evaluated the use of the Patient Global Assessment of Disease Activity Visual Analog Scale, a patient reporting tool used to measure pain in patients with rheumatoid arthritis. Given that pain is a symptom observed across all diseases, we believe these findings can be extended beyond the measurement of arthritis pain. This complex assessment tool is oftentimes confusing for LEP patients, and the lack of proper translation or help with navigation leads to inaccurate captures of these patients’ experiences. Hirsh et al^14^ suggested several strategies to improve the use of this patient reporting tool that may be adopted to enhance other PRO tools for use in a range of diseases, including oncology. These strategies include ensuring that a validated version is available in the patient’s primary language and appropriate literacy level and capturing the information through nonwritten formats such as audio or electronic reporting. They also proposed providing patients with a standard explanation about the domains being queried and soliciting continuous input from diverse patient groups regarding the design and validation of PRO measures.

Findings from these focus groups were used to generate themes and hypotheses on potential barriers to quality care for patients on OCDT, including LEP patients. We are using these findings to improve care through the use of ePRO tools at DFCI, including the deployment of ePRO tools in Spanish (completed in February 2020) and other languages (ongoing since 2022), and including systematic screening for social determinants of health and resource matching for the entire population (to be implemented in Fall 2022).

### Limitations

These findings should be interpreted in the light of their limitations, given that this was a single-site study with Spanish- and English-speaking patients. To better inform the design of technology interventions, including ePRO systems, so that they meet the needs of LEP patients, future studies should address LEP patients who speak other languages. Furthermore, we were unable to make direct comparisons between the EP and LEP focus groups because of the lack of LEP patients available to participate in comparison with the number of EP patients available to participate, although the distribution of LEP to EP focus groups approximates the distribution of LEP patients seen at DFCI.

## Conclusions

Although most interventions specifically target EP patients, our findings reveal the willingness of LEP patients to participate in technology-based interventions. Including LEP patients in interventions that leverage ePROs and other electronic tools may help to manage gaps in communication about treatment and potential adverse events because of the willingness of LEP patients to use ePRO tools to address these barriers. Despite the perceived difficulty of achieving language concordance in technology-based interventions and ePRO tools, we believe that inclusion of LEP patients is an invaluable enhancement of these efforts and could benefit equitable health care delivery.

## Notes

Supplement. **eAppendix.** Focus Group Guide
