# Supplementary material for: Barriers to Electronic Patient-Reported Outcome Measurement Among Patients with Cancer and Limited English Proficiency
Source: JAMA Netw Open. 2022 Jul 22;5(7):e2223898. doi: 10.1001/jamanetworkopen.2022.23898 (PMC9308052; doi:10.1001/jamanetworkopen.2022.23898)
Supplement: Supplement. — eAppendix. Focus Group Guide [file jamanetwopen-e2223898-s001.pdf]

Supplemental Online Content

Garcia Farina E, Rowell J, Revette A, et al. Barriers to electronic patient-reported outcome measurement among patients with cancer and limited English proficiency. *JAMA Netw Open.* 2022;5(7):e2223898. doi:10.1001/jamanetworkopen.2022.23898

eAppendix. Focus Group Guide

This supplemental material has been provided by the authors to give readers additional information about their work.

© 2022 Garcia Farina E et al. *JAMA Network Open.*

eAppendix. Focus Group Guide

Oral cancer directed therapy adherence focus group

Primary objective: Explore how patient's prescribed oral cancer directed therapy (OCDT) define treatment adherence
Secondary objectives:
Explore factors associated with OCDT adherence
Explore factors associated with lack of OCDT adherence

Introduction and Verbal Consent
Good evening and thank you for taking the time to be part of this focus group session [introduce moderator] Our goal is to better support patients that have been prescribed oral cancer directly therapy. As a first step, we want to better understand how patient's prescribed oral cancer directed therapy (OCDT) think about and approach their treatment and treatment adherence. You are part of this focus group because you have been prescribed oral cancer directed therapy for your cancer, and your opinion is very important for us. The information from this discussion will be used to help develop an OCDT self-management tool.

Our session will last approximately ninety minutes. As noted in the email, this session is being recorded because we don't want to miss any of your comments. This also allows me to focus on what you are saying rather than taking notes. We will be on first name basis and your comments will be anonymous. There will be no names attached to comments in our reports, we will not share your comments directly with your doctors, and it will not negatively impact your current clinical care. We also ask each of you to keep everything that is discussed today in this room confidential. I also request that each of you please turn off your cell phones so that the session is not interrupted.

My role as the moderator is to guide the discussion by asking questions and listening. There are no wrong answers and we do encourage you to discuss differing points of view. Please feel free to share your point of view even if it is different from what others have said. While it is OK to disagree with each other, please be respectful towards your fellow group members It is also important for us to hear from each of you today because you have different experiences. Therefore, if one of you is sharing a lot, I may ask you to let others talk. And if you are not sharing, I may ask you for your opinion, but it is OK if there are topics you do not wish to discuss. To be respectful of your time and the task at hand, I may need to interrupt at times to move us on. Thank you for your cooperation if those circumstances arise.

© 2022 Garcia Farina E et al. *JAMA Network Open.*

I'd like to start today by getting a better sense of your overall experience with taking OCDT

How would you describe your oral cancer directed therapy to someone unfamiliar with it?

What is your understanding of the goal of the OCDT?

What were your expectations when you were first prescribed OCDT?

What, if any, questions did you have for your care team about OCDT?

What, if anything, do you like about the idea of taking OCDT?

What, if anything, do you dislike about the idea of taking OCDT?

How have your experiences compared to your expectations around taking OCTD?

[We have discussed XYZ, can you describe any additional aspects of taking OCDT that are difficult?

How does OCDT compare to other treatment that you have had in the past?

[probe on IV drugs, side effects, importance of taking it as prescribed]

What is your understanding of what OCDT is doing as it relates to your medical/cancer care?

How important, if at all, is it for you to understand how it works in relation to your cancer?

What, if anything, is most important for you to understand about OCDT?

Now I'd like to focus on how you approach your schedule for taking OCDT

Can you walk me through your overall schedule for taking OCDT?

How you decide when
how
where to take OCDT?

How does your approach/schedule for taking OCDT compare to the way that your care team wants/expects you to take you to take it?

[As appropriate] What factors play the biggest role in you taking OCDT on your schedule verses as prescribed by your doctor?

How easy or difficult is it to remember to take OCDT?

How often to you remember/forget?

How do you feel (emotionally) when you forget to take OCDT?

What do you do when you remember or forget?

Do you tell anybody\y on your care team if you forget to take your OCDT?

© 2022 Garcia Farina E et al. *JAMA Network Open.*

Medication adherence is defined by the World Health Organization as "the degree to which the person's behavior corresponds with the agreed recommendations from a health care provider." Though the terms adherence and compliance are synonymously used adherence differs from compliance. Compliance is the extent to which a patient's behavior matches the prescriber's advice. Compliance implies patient obedience to the physician's authority, whereas adherence signifies that the patient and physician collaborate to improve the patient's health by integrating the physician's medical opinion and the patient's lifestyle, values and preferences for care.

Based on this definition of adherence: how much of how little do you adhere to your regimen?

From your perspective how important or unimportant is it to adhere to the OCDT regime as prescribed by your doctor?

What factors, if any, lead you to alter your adherence?

What, if any, do you see as the consequences of not adhering to your regimen as prescribed?

Do you think about what you are eating/drinking around taking your OCDT?

How do food/beverages play a role, if any, if your taking the OCDT?

Tell me about the information found on the labels of OCDT.

Is that something that you read/pay attention to?

What things are clear/unclear? Easy/difficult about the labels?

What, if anything, did your care team tell you about the label information?

How would you describe your care team's understanding of your experience with OCDT?

Is there anything your doctor doesn't understand about OCDT?

Can you walk me through the factors that determine when and why you tell you care team concerns or questions you have about OCDT?

For example, have you ever been hesitant to call your clinician about issues or concerns you have related to your OCDT?

What other things are important, from your perspective, when if comes to taking the OCDT?

What types of things would you tell a new patient about taking OCDT?

Now I would like to take some time to explore a program that is focused on adherence around OCDT.

First, I would like to better understand how, if at all, you typically use technology in your everyday life?

(probe on comfort/familiarity with smartphone, tablet, computer etc.)

Does anyone of any examples of how they use technology for their health or health information?

(probe as needed)

Have you ever used reminders/alarms to help remember to take medicines or keep a health schedule?

Apps?

What did you like/dislike?

© 2022 Garcia Farina E et al. *JAMA Network Open.*

I'd like to get your reaction to the idea of a mobile application to help improve your adherence to OCDT;

How do people feel about that idea?
What questions would you have?
What concerns do you have?
What features would you like it to have/avoid?

Program [Use Attached Handouts]

Which item did you select for your #1?
What made that a priority for you?
Which item did you select for you #7?
What made that a priority for you?

What other aspects of a self-management tool would you add to the list? (for example, A messaging app to communicate with and receive communications from your care team? Would you like to be notified about updates to your medical record or new messages via a text message?)

Anything else about a self-management app that we have not discussed?

DFCI is trying to create an adherence program that incorporates the self-management tool. Do you have any initial reactions to that idea?

[Explore patient willingness to participate in an adherence program if not yet discussed]

What factors would play a role in determining if you would participate in this program?

How would you prefer to be educated/trained on participating in the program? *(Education/training required to participate in adherence program (in-person, embedded within app, video upon enrollment)*

How often would you be willing to report? What would be most acceptable to you? *(daily, weekly, prn (e.g. When dose missed))*

How/what format would be preferred for reporting XYZ? *(call, smartphone, tablet, desktop integrated into calendar*

What types of things might motivate you to participate in a study like this?

What types of things would make you uninterested in a program like this?

We'd also like to include a monitoring program that looks at toxicity/side effects of OCDT,

© 2022 Garcia Farina E et al. *JAMA Network Open.*

[Discuss any aspects of symptom handout not already discussed]

What factors would play a role in determining if you would participate in this program?

How would you prefer to be educated/trained on participating in the program?
(Education/training required to participate in adherence program (in-person, embedded within app, video upon enrollment)

What frequency of reporting would be most acceptable to you? *(daily, weekly, prn (e.g. When dose missed))*

How/what format would be preferred for reporting XYZ? *(call, smartphone, tablet, desktop)*
What types of things might motivate you to participate in a study like this?

What types of things would make you uninterested in a program like this?

Explore utility of self-management tools to address reported toxicity or improve OCDT adherence

Is there anything else about OCDT that you would like to share before we finish our conversation today?

© 2022 Garcia Farina E et al. *JAMA Network Open.*
